# Risk strata-based therapy and outcome in stage Ib–IIa carcinoma cervix: single-centre ten-year experience

**DOI:** 10.3332/ecancer.2013.341

**Published:** 2013-08-20

**Authors:** Rajshekar S Kundargi, B Guruprasad, Praveen Shankar Rathod, PN Shakuntala, K Shobha, VR Pallavi, K Uma Devi, UD Bafna

**Affiliations:** 1 Department of Gynaec-oncology, Kidwai Memorial Institute of Oncology, Bangalore, Karnataka 560029, India; 2 Department of Medical Oncology, Kidwai Memorial Institute of Oncology, Bangalore, Karnataka 560029, India; 3 Department of Gynaec-oncology, Kidwai Memorial Institute of Oncology, Bangalore, Karnataka 560029, India

**Keywords:** carcinoma cervix Ib–IIa, radiotherapy, chemotherapy

## Abstract

**Aim:**

To review the outcome of stage (Ib, IIa), cervical cancer patients were primarily treated with radical hysterectomy and risk-based postoperative therapy.

**Material and methods:**

Between January 2001 and December 2011, 601 cases underwent surgery followed by tailored therapy. Patients were classified into low risk (pelvic lymph node negative, tumour less than 4 cm, no evidence of lympho-vascular invasion, less than one-third of thickness of surgical stoma involved), intermediate risk (positive lympho-vascular space invasion, tumour size more than 4 cm, and deep invasion of cervical stroma), and high risk (pelvic lymph node involved, positive parametrial, or vaginal margins) groups. Postoperative adju-vant therapy in the form of radiotherapy alone to those with intermediate risk and chemo-radiotherapy to those with high risk was given to patients. The median follow-up was 60 months.

**Results:**

The majority of patients had intermediate risk. The overall event-free survival (EFS) at five years was 74.37%, with EFS of 86.5% in those from the low-risk group, 73% in those from the intermediate-risk group, and 64% in those from the high-risk group. In conclusion, risk strata-based adjuvant postoperative therapy is able to provide a favourable outcome in patients with stage Ib–IIa cervical cancer with a nearly 11% improvement in survival compared with historical control.

## Introduction

The incidence of cervical cancer in developing countries remains nearly twice that in developed countries [1]. Early-stage cervical cancers (Ib, IIa) are treated with either radical hysterectomy and pelvic lymphadenectomy or chemoradiation. The reported overall survival rates after these procedures range close to 80%–90% in world series [2]. Historical data from the Indian scenario suggest that overall survival in the early stages is 63.3% at five years [3]. However, with the incorporation of risk-directed postoperative therapy in the last decade, the outcome may have improved. Although there is ample data in the world literature about this disease and outcome based on risk stratification, there is a dearth of data in our setting. Hence, the present study was undertaken.

## Material and methods

Six hundred and one cases of stage Ib–IIa carcinoma cervix, registered from January 2000 to December 2011 at a tertiary care cancer centre in South India, comprised the study group. The case records of these patients were analysed in detail for demographic profile, clinical features, treatment given, and outcome. All patients were clinically staged according to the International Federation of Gynecology and Obstetrics stag-ing system. These patients were subjected to radical hysterectomy with pelvic lymphadenopathy. Postoperative histopathology reports were reviewed, and postoperative complications were also noted. Patients were classified into low risk (pelvic lymph node negative, tumour less than 4 cm in size, no evidence of lympho-vascular invasion, and less than one-third of thickness of surgical stoma involved), intermediate risk (positive lympho-vascular space invasion, tumour size of more than 4 cm, and deep invasion of cervical stroma), and high risk (pelvic lymph node involved, positive parametrial or vaginal margins). Postoperative adjuvant therapy in the form of radiotherapy alone in those with inter-mediate risk and chemo-radiotherapy in those with high risk was given to patients.The radiotherapy that was used was given as external beam radiotherapy 45 Gy in 25# followed by brachytherapy (high dose rate or low dose rate). The concurrent chemotherapy was platinum-based (cisplatin 40 mg/m^2^) therapy given weekly. The patients were followed up with every three months for the initial two years and every six months thereafter to monitor for any recurrence of disease. The outcome was correlated with risk strata, histopathological type, tumour grade, and type of therapy given. In the intermediate-and high-risk groups, palliative chemotherapy with platinum and taxanes was given on recurrence. In the low-risk group, chemotherapy and radiotherapy were tried on recurrence whenever possible; otherwise only palliative chemotherapy was given. The outcome was evaluated for all patients using the Kaplan–Meier curve (SPSS 19 – SPSS Inc., USA).

## Results

The characteristics of patients are given in Tables 1 and 2. The median age of presentation was 49 years (range 22–80 years) in this retrospective study. The most common presenting symptom was white vaginal discharge, followed by the combination of bleeding and discharge, with 70% of them presenting within one to six months after the start of symptoms. Most patients were postmenopausal and multiparus. The majority had exophytic lesions with grade 3 squamous histology. These patients underwent radical hysterectomies (type 3) with no postoperative complications in 98% of the patients. The most common post-op complication was bladder atony, which was seen in 0.5% of the cases. Fifty-one percent had stage IBI. The median dose of external beam radiation therapy given was 48 Gy (range 34–58 Gy), and the median time required for the same was 40 days (35–55 days). In the risk stratification, most patients had intermediate risk strata. Overall event-free survival (EFS) at a median follow-up of five years was 74%. Correlating tumour histology and tumour grade with outcome, histology (adeno or squamous) did not correlate with survival; five-year EFS with grades 1, 2, and 3 was 87.5%, 75%, and 69%, respectively; however, this was not statistically significant (p = 0.8). Correlating each risk factor, EFS with lymph node positive (92% in not involved versus 71% in involved), parametrial invasion (without 94% versus with 58%), margin positivity (without 88% versus with 60%), and size > 4 cm (without 82% versus with 63%). A total of 150 events were noted on follow-up; of these only 83 received any form of therapy (chemoradiotherapy in 65% and chemotherapy alone in 35%), while others were lost to follow-up. Most recurrences occurred within four years, with the majority in the pelvis (65%).

**Figure 1:**
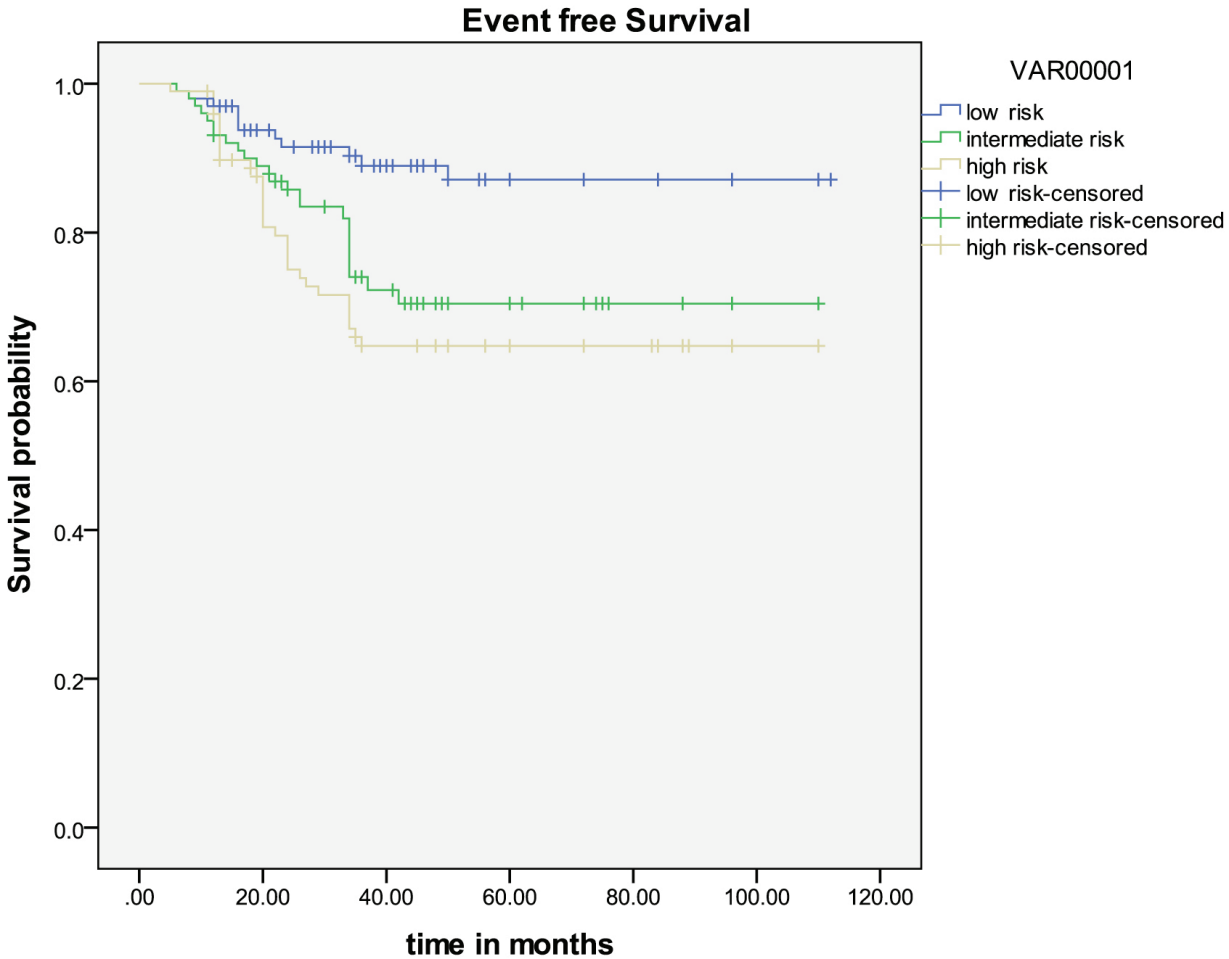
Event-free survival.

The overall EFS rate at five years was 74.37%, with EFS of 86.5% in low-risk patients, 73% in intermediate-risk patients, and 64% in the high-risk group (Figure 1).

## Discussion

In developing countries, cervical cancer is the second most common cancer among women [1]. India has one of the highest incidence rates of cervical cancer with poor survival rates [3]. To the best of our knowledge, this is the largest study of tailored postoperative therapy in India.

Many factors can influence survival in patients with stage Ib–IIa. These include lymph node status, parametrial invasion, depth of stromal invasion, size of tumour, surgical margins, lympho-vascular space invasion, and tumour grade [4–8]. Comparing our study with other studies, the median age, incidence of squamous histology, stage 1B tumours, stromal invasion, pelvic node positivity, and tumour size of more than 4 cm were similar to the world literature (Table 2). Nevertheless, we noted a lower incidence of parametrial and lympho-vascular invasion, and a higher incidence of grade 3 tumours.

The outcome of patients with each of the parameters ranges from 80%–95% when none of them are present to 50%–70% when any of the risk factors are present [4–8]. In the last decade, the prognosis has improved with risk-based treatment. With the use of radiotherapy alone in nodal positivity, the survival rate was 45%–55%, while in node negative, it was 85%–95% at five years. With the use of chemora-diotherapy, this has improved to 81% in node positive [9]. While previous studies showed that adenocarcinoma had poor survival compared with squamous histology, newer studies have not shown such trends [8, 10]. With more insight into the disease, even grade of tumour has not shown any increased risk of recurrence [11]. In our study, node-positive tumours had an EFS of 71% at five years, with no difference in outcome in adenocarcinoma histology and tumour grade. Patients without lymph vascular space invasion have a disease-free survival rate of 89%, which drops down to 77% when involved [12]. In our study, it was 92% without invasion versus 73% when involved. The survival rate reduces with parametrial involvement, increasing stromal invasion, margin positivity, and increasing size of tumour [13, 14]. This has been shown in our study as well.

Taken together, these factors are grouped into low, intermediate, and high risk. High risk includes lymph node metastases, positive surgical margin, and parametrial extension [9]. Intermediate risk includes >one-third stromal invasion, lympho-vascular space invasion, and tumour size > 4 cm [15]. All patients with none of the above-mentioned risk factors are considered low risk. The EFS in postoperative high-risk patients with chemoradiotherapy was 80% at four years in the initial study done by Peters *et al* [9]; in our study, this was 64% at five years (Figure 1). In the postoperative intermediate-risk group, the EFS with radiotherapy alone was 88% [15]; this was 73% in our study. A review of the literature shows that in low-risk patients, the EFS at five years with surgery alone is 90%; this was 86.5% in our study [16]. Hence optimum results seem to be achievable even in our setting with radical hysterectomy followed by postoperative, risk-stratified adjuvant therapy.

When comparing this with historical control of 2,121 patients diagnosed and treated during 1982–1989, there is an improved EFS of nearly 11% (74.37% versus 63.3%) [3]. Back then, the role of chemotherapy was limited to advanced cancer, and patients in the high-risk group did not receive any additional therapy. Hence tailored adjuvant therapy is able to obtain a satisfactory clinical outcome in patients with early-stage cervical cancer.

With definitive therapy, 30% of patients will recur; the treatment of these will depend on the primary therapy received, pattern of relapse, and performance status of patient [17]. The recurrence pattern was similar to other larger studies [18].

In conclusion, risk strata-based adjuvant postoperative therapy is able to provide a favourable outcome in patients with stage Ib–IIa cervical cancer. With the availability of HPV vaccines, newer modalities of therapy like neo-adjuvant therapy, and the use of molecular markers, the future of cervical cancer even in the Indian scenario looks very promising.

## Figures and Tables

**Table 1:** Demographic profile.

Age of presentation	
<50 years	62.6%
50–69	34%
>70	3.4%
Symptoms	
Vaginal discharge	41%
Vaginal bleeding	24%
Both	35%
Duration of symptom	
<One month	69.6%
One to six months	19.4%
>Six months	11%
Premenopausal	40.1%
Postmenopausal	59.9%
Parity	
Primipara	3.8%
Multiparous (p2, p3, p4, p5, p>5)	96.2% (20.5%, 28%, 19.1%, 13.1%, 15.5%)
Age at marriage	
<15 years	23.5%
16–20 years	71%
>20 years	5.5%
Tumour type	
Exophytic lesion	46.4%
Endophytic lesion	21.8%
Infiltrative lesion	31.8%
Complication of surgery	
Nil	98%
UVF	0.16%
VVF	0.16%
Bladder atony	0.5%
Ileus	1.1%
Number of patients in each group	
Low risk	26.3%
Intermediate risk	47.2%
High risk	26.5%

**Table 2:** Comparison of various studies.

Patient characteristics	Our study (*n*= 601)	Takeshima *et al*(*n*= 65)	Schorge *et al* [16](*n*= 171)	Landoni *et al* [4](*n*= 343)	Ho *et al* (*n*= 197)	Tsai *et al* (*n*= 222)
Median age (years)	49	45	46		47	50
Clinical stage						
IBI	68%	55%	79%	IB: 88%	IB: 82%	IB: 79%
IB2	20.4%	37%	12%			
IIA	11.6%	8%	9%	12%	18%	21%
Cell type						
Squamous	87%	78%	73%	83%	79%	88%
Adeno	10%	22%	16%	14%	14%	
Small cell	0.16%			3%		
Clear cell	0.16%				1%	
Adenosquamous	2.6%				6%	
Stromal invasion Present	70%	84%	42%		41%	
Pelvic nodes Positive	18.5%	47%		13%	23%	33%
Parametrial invasion	5.5%	12%			11%	21%
Positive margins	14%	8%	5%	11%	5%	12%
Tumour size > 4 cm	23%		14%	32%	25%	25%
Tumour grade: 3	63%		43%	46%	41%	
Lymphovascular invasion	17%	69%	45%		39%	48%
